# Using Behavioral Nudges to Engage Pregnant Women in a Smoking Cessation Trial: An Online Field Quasi-Experiment

**DOI:** 10.3390/healthcare8040531

**Published:** 2020-12-02

**Authors:** Oana M. Blaga, Teodora D. Frățilă, Cristian I. Meghea

**Affiliations:** 1 Center for Health Policy and Public Health, College of Political, Administrative and Communication Sciences, Babeș-Bolyai University, 400376 Cluj-Napoca, Romania; teodora.fratila@publichealth.ro (T.D.F.); meghea@msu.edu (C.I.M.); 2 Department of Public Health, College of Political, Administrative and Communication Sciences, Babeș-Bolyai University, 400376 Cluj-Napoca, Romania; 3 Department of Obstetrics, Gynecology and Reproductive Biology, College of Human Medicine, Michigan State University, A134 East Fee Hall, East Lansing, MI 48824-1316, USA

**Keywords:** behavioral nudges, randomized controlled trials, enrollment

## Abstract

Evidence shows that behavioral nudges could be used to enhance enrollment rates in randomized controlled trials (RCTs) by addressing enrollment barriers, but research on this topic is limited. We conducted an online field quasi-experiment with separate pretest (October 2017–January 2018) and posttest (February–May 2018) samples designed to examine the use of behavioral nudges to engage pregnant smokers in a couple-focused smoking cessation RCT relying on online enrollment through paid Facebook ads and a dedicated website, by reporting aggregate Facebook ads and Google Analytics data. The Facebook ads pretest conversion rate of 1.6% doubled and reached 3.41% in the posttest period. The pretest eligibility assessment rate decreased from 10.3% to 6.46%, but registered a relative increase of approximately 50% in the posttest period, as opposed to the pretest. The number of women who signed the informed consent in the posttest period has increased with 63%, from a proportion of 8.54% in the pretest to 11.73% in the posttest period. These findings might lend support to integrating behavioral nudges in the recruitment and enrollment materials of RCTs to boost enrollment.

## 1. Introduction

Many randomized trials (RCTs) fail to meet their recruitment targets [1] and participant recruitment represents one of the main challenges of RCTs [2]. This translates into increased trial costs and failure to reach adequate statistical power [3,4]. Challenges in recruiting human subjects can be related to participant characteristics (such as demographic characteristics), contextual factors (i.e., culture or political environment), as well as research-related factors (i.e., complex research protocols, random assignment) [2]. Recent evidence shows that behavioral nudges could be used to enhance RCT enrollment rates by addressing enrollment barriers [5]. For example, two studies that made use of behavioral nudges in the form of personalization [6] and message framing [7] found them effective in increasing enrollment rates in an RCT of screening of undiagnosed atrial fibrillation and in an RCT designed to test different strategies to increase willingness to undergo research lumbar puncture. Behavioral nudges have been defined as any “aspect of the choice architecture that alters people’s behavior in a predictable way without forbidding any options or significantly changing their economic incentives” [8]. Yet, the research on this topic is limited, with most focusing on the potential ethical concerns raised by using behavioral nudges to obtain individuals’ informed consent for research enrollment [9,10].

In this exploratory study, we aimed to investigate whether behavioral nudges can improve participants’ recruitment in the Quit Together (QT) couple-focused pregnancy smoking cessation RCT [11]. The RCT was funded to develop and pilot test a smoking cessation intervention with a focus on couples, and to build research capacity in Romania, a low- and middle-income country at that time. Other reasons why the RCT was implemented in Romania included the relatively high pregnancy-smoking rate and the fact that virtually all of the pregnant women in Romania are married or live with a partner [12], which made the couple-focused intervention more feasible.

We employed the behavioral diagnosis and design framework [13] as the blueprint of our online quasi-experiment, described in more detail below. The use of this framework implies that the program or intervention is already being delivered and participant data are available for analysis to assist in developing appropriate behavioral solutions to increase enrollment [13].

This paper examined whether the use of behavioral nudges to inform the development of trial recruitment paid Facebook ads and a modified interface for its website has influenced: (1) the Facebook ads conversion rate (percentage of people who saw the ad vs. who clicked om the ad and reached the website); (2) the percentage of individuals who reached the website and who initiated the process of self-assessing their eligibility to participate in the RCT; and (3) the proportion of women who signed the informed consent among those who initiated the self-enrollment process.

## 2. Materials and Methods

### 2.1. Study Design

We conducted a field quasi-experiment with separate pretest and posttest samples. Aggregate data were collected through Facebook Ads Manager and Google Analytics, for 93 days, between 22 October 2017–22 January 2018 (pretest data) and 93 days, 20 February–24 May 2018 (posttest data). The Quit Together RCT was implemented in Romania and consisted of over-the-phone motivational interviewing counselling sessions, designed to increase motivation and facilitate problem solving related to difficulties pregnant women meet in their smoking cessation attempts. The RCT protocol has been described elsewhere [9]. In brief, the enrollment process involved online promotion through paid Facebook ads and a dedicated project website with direct links to RCT forms hosted on the secured Qualtrics [14] platform. Facebook was used as recruitment source in the quasi-experiment proposed in this paper because it was also used to recruit participants in the larger RCT the quasi-experiment was nested in. The paid Facebook ads were set up to target women aged 18–40, who were living in Romania, and had interests in pregnancy, motherhood, and tobacco use. These population parameters and the Facebook weekly ads budget ($24/week) remained unchanged in the pretest and posttest periods, which were also identical (93 days each). Website visitors interested in RCT participation filled out an eligibility assessment form, an electronic consent form, and a baseline survey. RCT eligibility criteria included being 18 or older, being pregnant, a current smoker, and willingness to offer their own contact information and the contact details of the partner. Enrollment in the trial began in June 2017, six months prior to the implementation of the experiment described in this paper.

This field quasi-experiment involved the natural selection of participants based on their performance of natural or everyday tasks (i.e., the action of clicking the project’s Facebook ad due to their interest in the topic of the ad). The participation in this experiment did not involve the subject’s authentication or identification. This type of experiment implies that subjects do not know they are participating in a research study. Hence, in nearly all cases, subjects are not asked to sing an informed consent [15], as this can influence their behavior due to their awareness of being studied [16]. Due to these reasons, no informed consent was requested from participants before clicking on the Facebook Ad or when browsing the QT website. This quasi-experiment was run before the General Data Protection Regulation went into effect in Romania, on 25 May 2018. Any participants eligible to participate in the RCT at the end of the self-conducted eligibility assessment were presented with the RCT’s informed consent form, approved by the Institutional Review Board at the Michigan State University (IRB# 14-910).

### 2.2. Population and Sample

The intent of the Facebook ads was to target pregnant smokers in Romania who have access to the internet and own a mobile device able to connect to the internet, which was the RCT target population. The sample of the quasi-experiment consisted of 124,359 RCT enrollment Facebook ad views (50,970 ad views before the experiment, and 73,389 after the experiment).

### 2.3. The Quasi-Experiment Designed to Increase Engagement with the RCT

**Figure 1:**
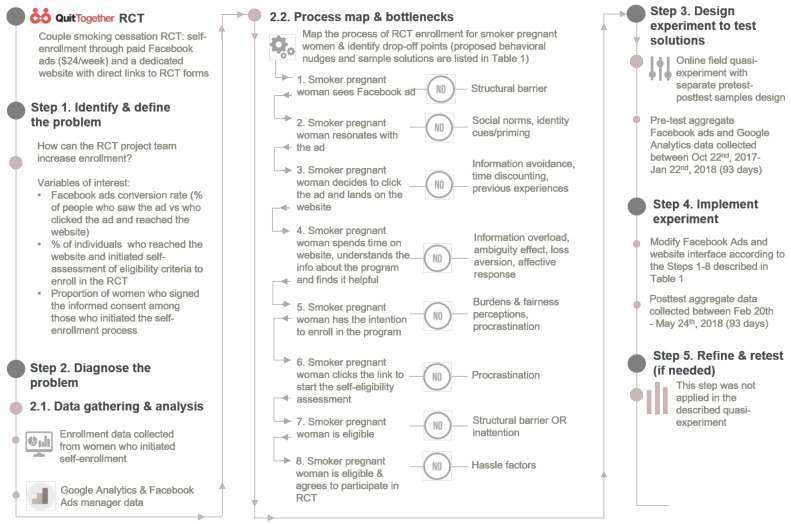
The application of the Behavioral diagnosis and design framework [13] to the quasi-experiment designed to increase enrollment in the Quit Together (QT) randomized controlled trial (RCT).

The quasi-experiment was designed following the five steps of the behavioral diagnosis and design framework [13]. More specifically, the five steps of this framework—problem definition, data collection to inform the development of a behavioral process map, design of behavioral solutions/nudges, selection of an of appropriate study design to test behavioral nudges, and refining and re-testing—were used to guide the development of our intervention and the implementation of this quasi-experiment. The application of the behavioral diagnosis and design framework to our quasi-experiment is visually described in Figure 1. In order to identify the points in the enrollment process that hinder participants to access the project’s website, and to initiate the eligibility criteria screener, we reviewed and analyzed: (1) the enrollment data collected from women who already initiated self-enrollment (i.e., time spent to fill out the self-eligibility assessment form); and (2) the aggregated Facebook Ads manager and Google Analytics data collected in the first six months of the trial (i.e., time spent on RCT’s website, users’ behavior on the website). For example, Google Analytics data showed that the average time spent on the website in the first six months of recruitment was 35 s per visit, with most visitors (93%) spending less than 10 s per website visit session. This suggested that the landing page was not user friendly and did not present information in a way that would make the user spend time on the website to learn more about the smoking cessation program.

The identification of behavioral nudges to increase engagement with the QT RCT was conducted by the first author, following a rigorous and systematic three-step process: (1) identifying specific points/stages in the enrollment process through which a potential participant (smoker pregnant woman) should go in order to enroll in the RCT; these points map the enrollment process; (2) identifying behavioral biases or bottlenecks potentially associated with each stage of the enrollment process; these constitute potential drop-off points for participants; and (3) proposing potential behavioral nudges as solutions designed to prevent drop-offs at each step of the enrollment process. The process map for QT RCT enrollment, potential drop-off points, bottlenecks, behavioral nudges/solutions, and sample materials are available in Table 1. All identified solutions were implemented before the posttest assessment.

The list of identified behavioral biases or bottlenecks that might affect the engagement of potential participants with the Facebook Ads and website of the trial included information overload [17], anchoring heuristics [18], ambiguity effect [19], framing [20], inattention [21], information avoidance [22], loss aversion [23], or time discounting [24]. The solutions we proposed and implemented to counteract the biases are informed by behavioral nudges and are presented in Table 1. For example, in order to tackle information overload, once users landed on the RCT website, we adjusted the content of the website by simplifying the available information and reduced the ambiguity by including an infographic designed to present the steps of the enrollment process. The before and after versions of RCT’s Facebook Ads and web interface are available as Supplementary Material 1. The first author created the content of the Facebook ads and the website and the second author posted the ads and made the changes to the website interface.

### 2.4. Data and Measurements

For this quasi-experiment, aggregate Facebook Ads and Google Analytics data were available. The main outcomes of interest were: (1) the Facebook Ads conversion rate (percentage of ad views resulting in a click on the ad, with a potential RCT participant landing on the project’s website); (2) the percentage of individuals who initiated the process of assessing their eligibility to participate in the RCT among those who reached the project’s website; and (3) the proportion of women who enrolled in the RCT (by signing the informed consent) among those who initiated the self-enrollment process.

### 2.5. Data Analysis

This experiment made use of aggregate Facebook ads and Google Analytics data collected in the pretest and posttest periods. No individual-level data were used. We were not able to perform statistical tests between pre-post intervention group because the available data were aggregated (i.e., total counts and percentages from Facebook and Google Analytics) and no sample variances were available. Thus, data analysis includes comparisons of absolute numbers and percentages on the variables of interest (Table 2).

## 3. Results

When comparing identical pre- and post-experiment ad campaign durations (93 days) with the same weekly campaign budget, there were 50,970 ad views pre-experiment and 73,389 post-experiment, a 30% increase in ad views (Table 2). The ad conversion rate, the percentage of individuals who clicked on ads and landed on the RCT enrollment website, more than doubled (1.56% vs. 3.41%) in the posttest period as opposed to the pretest period. The proportion of individuals who initiated the self-assessment of their eligibility among those who reached the project’s website decreased from 10.3% in the pretest period to 6.46% in the posttest period. In addition, the proportion of pregnant smokers who enrolled in the RCT among those who initiated the self-assessment of their eligibility increased from 8.54% to 11.73%, with almost three times more subjects getting enrolled in the RCT as opposed to the pretest period (7 vs. 19).

## 4. Discussion

The main finding of this study is that the Facebook ad conversion rate has more than doubled following the use of behavioral nudges in the development of Facebook ads, while using identical parameters for population ad targeting, the same weekly budget for Facebook ads, and with identical ad campaign durations in both pre-and posttest period. At the same time, the eligibility assessment rate decreased following the behavioral nudge experiment. It is possible that adjustments of the website interface made the information about the RCT easier to understand and, consequently, women who were not eligible to participate were able to understand this from the information listed on the website and were less likely to initiate the self-assessment of their eligibility post-experiment. Importantly, the proportion of women who enrolled in the RCT increased by almost two-thirds, resulting in close to three times more women enrolled post-experiment vs. pre-experiment.

Strengths of this quasi-experiment include the fact that it takes place in a natural setting for the study population, resembling the real-life situation in which pregnant female smokers, who are interested in quitting smoking, see an ad promoting a smoking cessation program on their Facebook news feed, and decide (or not) to find out more information about it. This real-life scenario increases the external validity of our results. Several of the more important study limitations merit attention. First, it is possible the results could be due to other factors (e.g., more ad views because of improved Facebook algorithm; changes in participants’ characteristics). However, it is likely that the large increase in the ad conversion rate and the increased percentage of enrolled subjects are due to the behavioral nudge experiment, because the parameters of the Facebook target population (age range, gender, interests), the duration, and the weekly ad campaign budgets were unchanged pre- and post-experiment. Second, due to availability of only aggregate data for our variables of interest (e.g., Facebook ads clicks at two points in time), the study authors did not perform statistical tests to assess the significance of the pre-post experiment changes and were not able to account for individual characteristics that may have influenced the results. Nevertheless, the aggregated findings do show a large, more than double, increase in the ad conversion rate, and an increase in the proportion and number of enrolled subjects following the implementation of the behavioral nudges. Third, due to the experiment being nested into an ongoing RCT, we could not manipulate the amount of exposure to or specific combinations of behavioral nudges. Future studies may use randomized factorial designs to gain greater insights into which changes (or cluster of changes) in Facebook ads and the RCT’s website interface have had the greatest impact.

Despite limitations such as these, the study findings are of interest because they provide initial evidence that might support the use of behavioral nudges to increase enrollment in RCTs and other types of research studies. There are few studies informing the topic in general, including one study providing a taxonomy of proposed behavioral economics interventions to improve clinical trial enrollment [5] and one discussing ethical aspects of using nudges [10]. Behavioral economics approaches have raised potential ethical concerns due to the adjustments the researchers might make to the choice architecture (i.e., changing default options; recruitment by physicians or authority figures) [9]. Since our experiment was limited to changes made to recruitment materials (Facebook Ads and RCT website interface) and it did not involve any changes in the consent procedures, it raised no ethical concerns. It is ethically defensible to use such behavioral nudges because they do not constrain in any way the subjects’ freedom of choosing to enroll in the RCT.

Very few studies used behavioral nudges as a tool to improve clinical trial enrollment. To the best of our knowledge, there are only two other studies published on this topic. Consistent with our results, an RCT of screening of undiagnosed atrial fibrillation that used behavioral nudges under multiple forms, including personalization, managed to increase enrollment rates from 0.8% to 9.4% over 10 months [6]. Similarly, Witbracht et al. [7] found that message framing was effective in changing subjects’ initial negative response to undergo research lumbar puncture, with participants randomized to the gain framed intervention having 67% higher odds of agreeing to be contacted about studies requiring lumbar puncture compared to those randomized to the loss frame.

Behavioral nudges represent low-cost solutions that have been proven to advance health, prevent diseases, and reduce morbidity and mortality [25]. They are considered low-cost solutions because they entail small, inexpensive changes, which require only few resources to be implemented. However, they generate large improvements in outcomes. Using behavioral nudges to increase recruitment in RCTs has some advantages compared to other strategies because they often involve small and inexpensive adjustments to the enrollment process, that could result in substantial changes in enrollment rates. In contrast, other strategies that have been found effective in increasing recruitment in RCTs require significant monetary and time resources to implement (from both researchers and study participants): telephone reminders or monetary incentives [26], an interactive computer program, attendance of an education session, or viewing a video about the health condition being researched [27].

## 5. Conclusions

The current study meaningfully contributes to the literature on using behavioral nudges to improve enrollment in research studies. The findings of this study provide initial evidence that might support the use of behavioral nudges to increase enrollment in RCTs and other types of research studies. An important issue for future research on this topic is to establish which specific behavioral nudges have more success in increasing enrollment rates, while minimizing potential ethical concerns.

## Figures and Tables

**Table 1:** Process map for smoker pregnant woman (SPW) enrollment in the QT RCT, possible drop-off points/bottlenecks, behavioral nudges, and sample intervention texts/materials.

Steps in the Enrollment Process & Possible Drop-off Points	Bottleneck	Behavioral Nudge/Solution	Sample Intervention Text/Material
1. SPW sees Facebook ad	No internet access	Not applicable, this is a structural barrier	Not applicable
2. SPW resonates with the Facebook Ad	Social norms	Enhance social motivators/social norms	Over 2000 future moms have already visited our website to learn more about the Quit Together program. Why not find out more about our free smoking cessation program?
	Identity cues/priming	Include pictures depicting pregnant women/couples and babies in Facebook ads, as well as testimonials of previous participants.	“The Quit Together program was the cornerstone of my quitting” Ana, aged 30–38 weeks pregnant.
3. SPW decides to click the ad and lands on the website	Information avoidance	Prompt contemplation Use affirmation	Why not find out more about this opportunity to quit smoking? It is important for you to have a healthy baby and you would do anything to for her/him. By quitting smoking, you are protecting your baby from many health conditions such as asthma and respiratory diseases.
	Time discounting	implementation prompts	What is your plan for quitting smoking?
	Previous experiences	Prevent SPW to rely on their experience with smoking during previous pregnancies and good pregnancy outcomes	Maybe you smoked in your previous pregnancy and you had a healthy baby. Since all pregnancies and babies are unique, you do not know how smoking will affect your current pregnancy. Find out more information about how you can quit.
4. SPW spends time on website, understands the information about the RCT and finds it helpful	Information overload	Adjust the website interface, simplify the available information, clearly explain the benefits of participating in the program (both economic & psychological, i.e., peace of mind).	It only takes 2 min to assess your eligibility. You’ll thank yourself later.
	Ambiguity effect	include an infographic designed to present the steps of enrolling in the program, the amount of time it would take, and what the benefits will be	Infographic is included in the Supplementary Materials
	Loss aversion	Emphasize benefits of participating	By not enrolling, you may: Miss out on 8 free counselling sessions with a counsellor specialized in… Lose up to 150 RON…
	Affective response	Adjust the website and its layout to induce a positive affect response	Use a handwritten post-it with a testimonial about the project.
5. SPW has the intention to enroll in the RCT	Burdens & fairness perceptions	Make the enrollment process as easy and straightforward as possible by letting women know all the time what is their next step to enroll in the project and how long it will take (normative feedback)	
	Procrastination	Sunk fallacy cost: provide an anchor for women by emphasizing the amount of time they will need to participate	It only takes two minutes to see if you are eligible. Think about how much time you lost today in traffic or doing grocery shopping. The two minutes can help you offer your baby a healthy start in life.
6. SPW clicks the link to start the self-eligibility assessment	Procrastination	Sunk fallacy cost	When visitors want to leave the website, set up a pop-up screen: “You already spent some time thinking about enrolling in this program. Why don’t you spend 2 more minutes to see if you would be eligible to actually participate?”
7. SPW is eligible to participate in the RCT	Structural barrier	The SPW does not meet the eligibility criteria	NA
	Inattention	Highlight eligibility criteria	Make more visible on the website’s landing page that: -partners’ smoking status is not an eligibility criterion -partners’ participation only takes 17 min of their time.
8. SPW is eligible and agrees to participate in RCT	Hassle factors	Describe the steps in the enrollment process	Use an infographic (included in the Supplementary Materials) to help women through the process. Mention that they can stop from filling out the form anytime and can return to finish it in a 1-week time span.

**Table 2:** Results of the online quasi-experiment (pre-post assessments using aggregate Facebook ads and Google Analytics data).

Variable of Interest	Pre-Experiment 93 Days $24/Week (*n* = 50,970 ad Views)		Post-Experiment 93 Days $24/Week (*n* = 73,389 ad Views)	
	*N*	%	*N*	%
Facebook Ads conversion rate	796	1.56	2506	3.41
Individuals who reached the project’s website and initiated the process of assessing their eligibility to participate in the RCT	82	10.3	162	6.46
Women who enrolled in the RCT assessed against the number of subjects who initiated the self-enrollment process	7	8.54	19	11.73

## Supplementary Materials

The following are available online at https://www.mdpi.com/2227-9032/8/4/531/s1, Supplementary material 1: Before and after versions of RCT’s Facebook Ads and web interface.

## References

[1] Gillies K., Cotton S.C., Brehaut J.C., Politi M.C., Skea Z. (2015). Decision aids for people considering taking part in clinical trials. Cochrane Database Syst. Rev..

[2] Gul R.B., Ali P.A. (2010). Clinical trials: The challenge of recruitment and retention of participants. J. Clin. Nurs..

[3] Sully B.G., Julious S.A., Nicholl J. (2013). A reinvestigation of recruitment to randomised, controlled, multicenter trials: A review of trials funded by two UK funding agencies. Trials.

[4] Emanuel E.J., Schnipper L.E., Kamin D.Y., Levinson J., Lichter A.S. (2003). The Costs of Conducting Clinical Research. J. Clin. Oncol..

[5] Vanepps E.M., Volpp K.G., Halpern S.D. (2016). A nudge toward participation: Improving clinical trial enrollment with behavioral economics. Sci. Transl. Med..

[6] Baca-Motes K., Edwards A.M., Waalen J., Edmonds S., Mehta R.R., Ariniello L., Ebner G., Talantov D., Fastenau J.M., Carter C.T. (2019). Digital recruitment and enrollment in a remote nationwide trial of screening for undiagnosed atrial fibrillation: Lessons from the randomized, controlled mSToPS trial. Contemp. Clin. Trials Commun..

[7] Witbracht M.G., Bernstein O.M., Lin V., Salazar C.R., Sajjadi S.A., Hoang D., Cox C.G., Gillen D.L., Grill J.D. (2020). Education and Message Framing Increase Willingness to Undergo Research Lumbar Puncture: A Randomized Controlled Trial. Front. Med..

[8] Thaler R., Sunstein C. (2008). Nudge: Improving Decisions about Health, Wealth, and Happiness.

[9] Mehlman M.J., Kodish E., Berg J. (2018). Ethical Issues in the Use of Nudges to Obtain Informed Consent for Biomedical Research. IRB Ethics Hum. Res..

[10] Cohen S. (2013). Nudging and Informed Consent. Am. J. Bioeth..

[11] Meghea C.I., Brinzaniuc A., Sidor A., Chereches R.M., Mihu D., Iuhas C.I., Stamatian F., Caracostea G., Dascal M.D., Foley K. (2018). A couples-focused intervention for smoking cessation during pregnancy: The study protocol of the Quit Together pilot randomized controlled trial. Tob. Prev. Cessat..

[12] Blaga O.M., Rus I.A., Wallis A.B., Brînzaniuc A., Cherecheș R.M. (2016). Smoking and Smoking Cessation during Pregnancy. An Analysis of a Hospital Based Cohort of Women in Romania. J. Community Heal..

[13] Richburg-Hayes L., Anzelone C., Dechausay N., Landers P. Nudging Change in Human Services: Final Report of the Behavioral Interventions to Advance Self-Sufficiency (BIAS) Project. www.mdrc.org/sites/default/files/2017_MDRC_BIAS_Final_Report_FR.pdf.

[14] Qualtrics (2016). Qualtrics. https://www.qualtrics.com/.

[15] Chen Y., Konstan J.A. (2015). Online field experiments: A selective survey of methods. J. Econ. Sci. Assoc..

[16] Couture P., List J.A. (2008). Informed Consent in Social Science. Science.

[17] Baddeley M. (2017). Behavioural Economics: A Very Short Introduction.

[18] Tversky A., Kahneman D. (1974). Judgment under Uncertainty: Heuristics and Biases. Sci. New Ser..

[19] Ellsberg D. (1961). Risk, Ambiguity, and the Savage Axioms. Q. J. Econ..

[20] Samson A., Barker L., Bright I. (2017). The Behavioral Economics Guide 2017 (with an Introduction by Cass Sunstein).

[21] Gabaix X. (2017). Behavioral Inattention. Handbook of Behavioral Economics: Applications and Foundations 1.

[22] Golman R., Hagmann D., Loewenstein G. (2017). Information Avoidance. J. Econ. Lit..

[23] Kahneman D., Tversky A. (1979). Prospect Theory: An Analysis of Decision under risk. Econometrica.

[24] Samson A. (2014). The Behavioral Economics Guide 2014. https://www.behavioraleconomics.com/be-guide/the-behavioral-economics-guide-2014/.

[25] Roberto C.A., Kawachi I. (2015). Behavioral Economics and Public Health.

[26] Watson J., Torgerson D. (2006). Increasing recruitment to randomised trials: A review of randomised controlled trials. BMC Med. Res. Methodol..

[27] Caldwell P.H.Y., Hamilton S., Tan A., Craig J. (2010). Strategies for Increasing Recruitment to Randomised Controlled Trials: Systematic Review. PLoS Med..

