# Supplementary material for: Using Behavioral Nudges to Engage Pregnant Women in a Smoking Cessation Trial: An Online Field Quasi-Experiment
Source: Healthcare (Basel). 2020 Dec 2;8(4):531. doi: 10.3390/healthcare8040531 (PMC7761597; doi:10.3390/healthcare8040531)
Supplement: Supplementary file 1 [file healthcare-08-00531-s001.pdf]

healthcare

MDPI

Supplementary Material S1: Before and after versions of RCT's Facebook Ads and web interface*

*The original text in these figures was in Romanian.

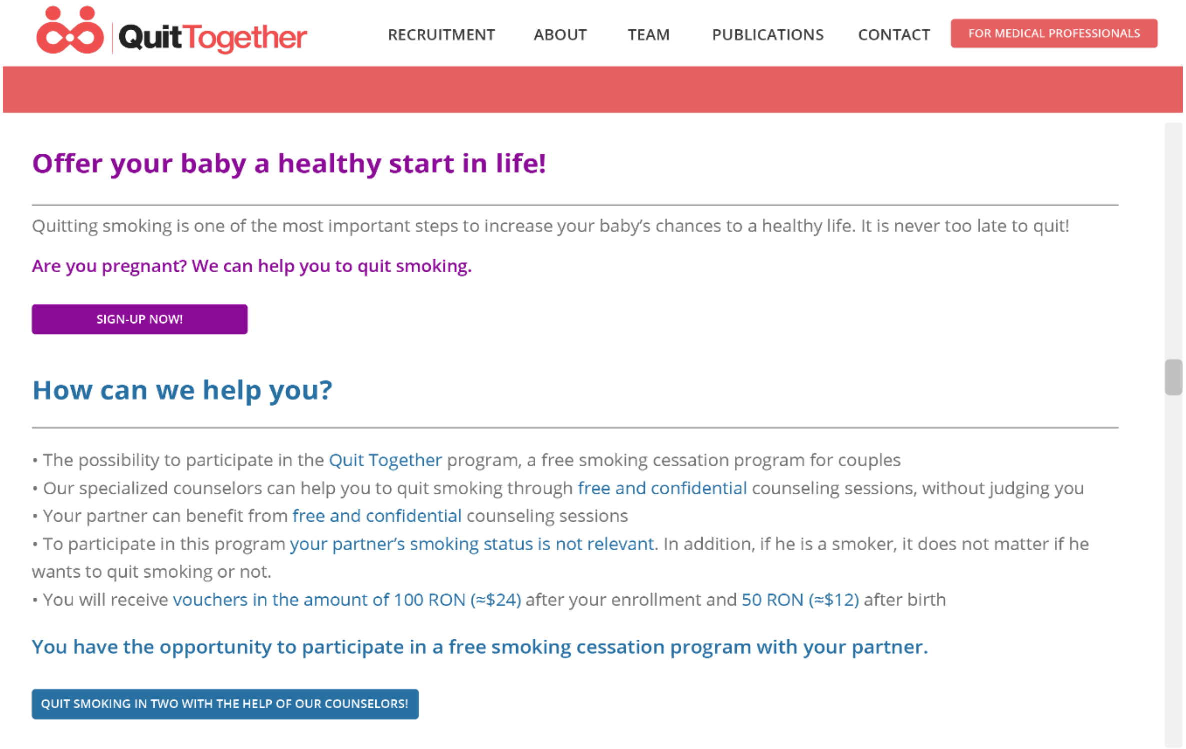

Figure S1. Screenshot of the Quit Together RCT's enrollment webpage depicting two value propositions.

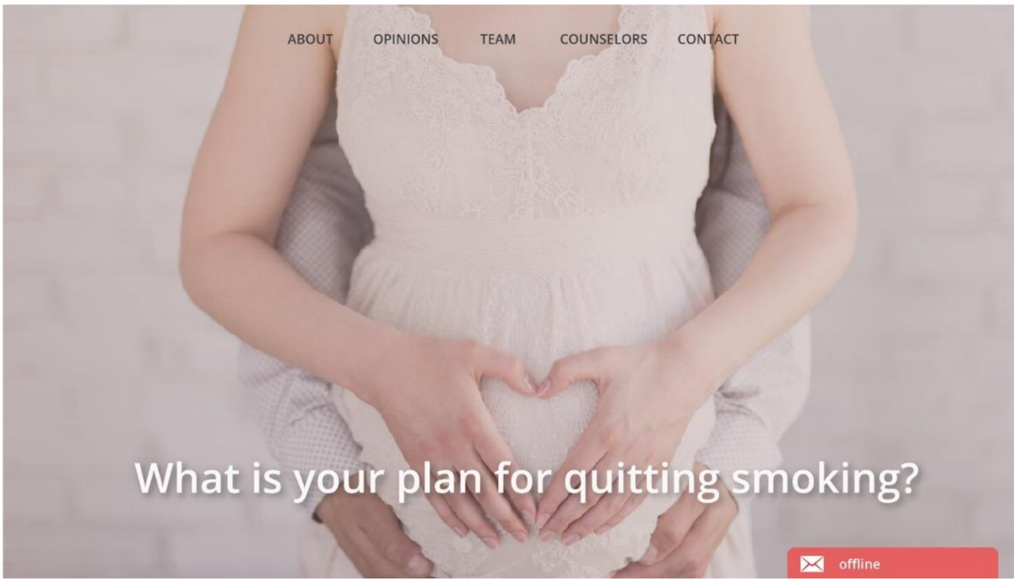

Figure S2. The new landing page of the Quit Together website (with live-chat feature included).

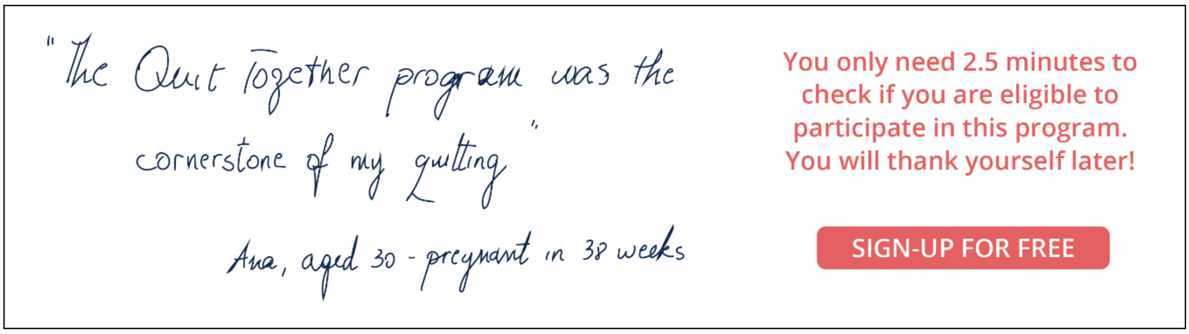

Figure S3. Testimonial of RCT participant and enrollment prompt posted on the Quit Together website.

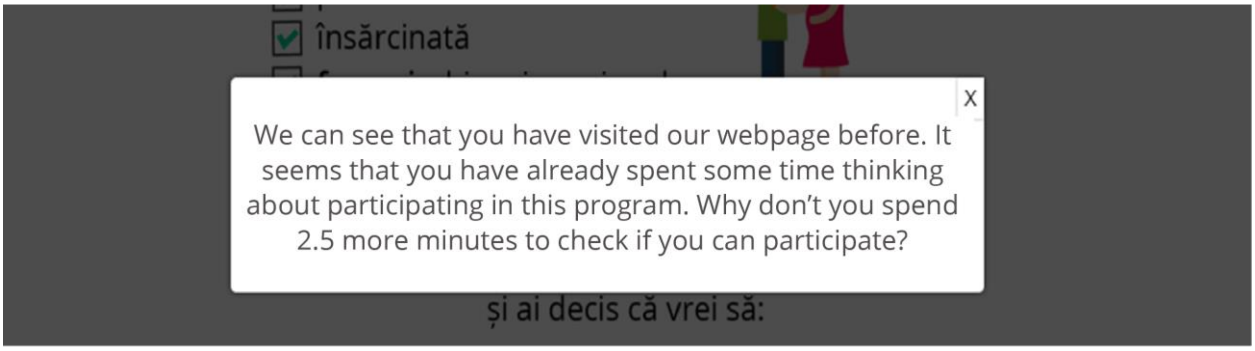

Figure S4. Pop-up message for returning visitors on the Quit Together website.

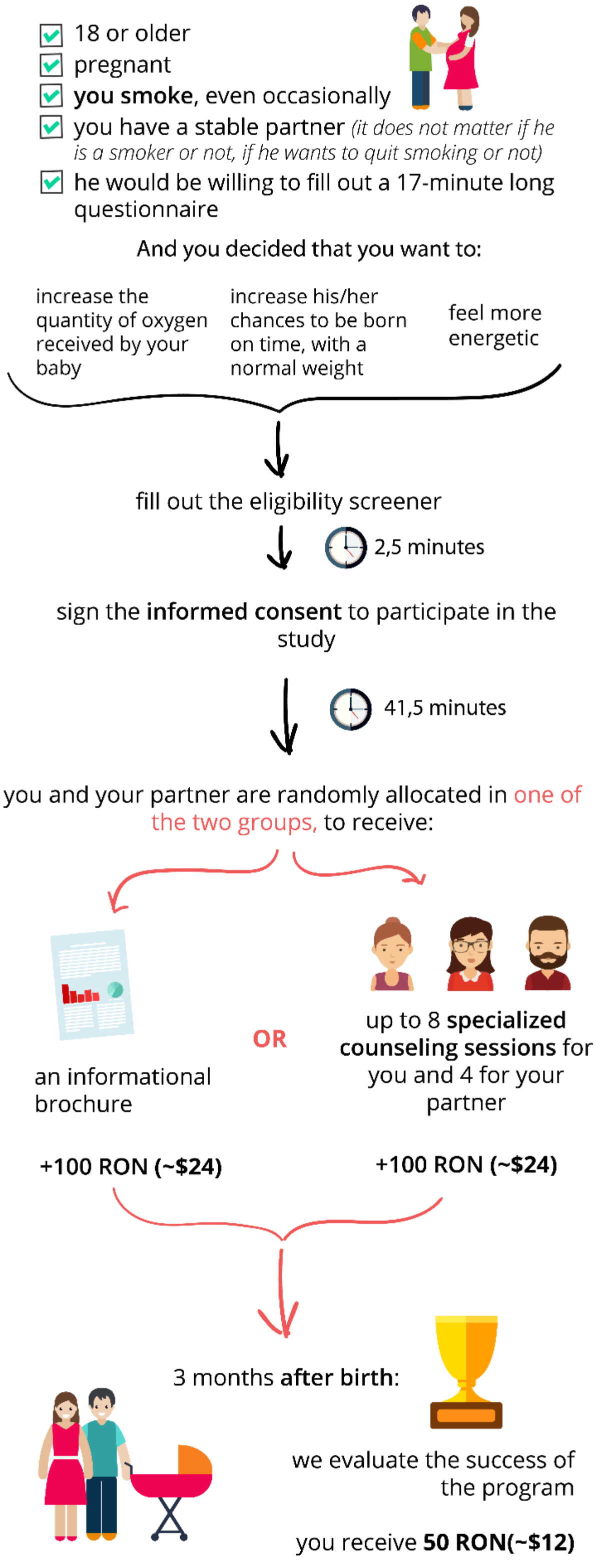

Figure S5. Infographic on the participation in the Quit Together RCT as posted on the website.

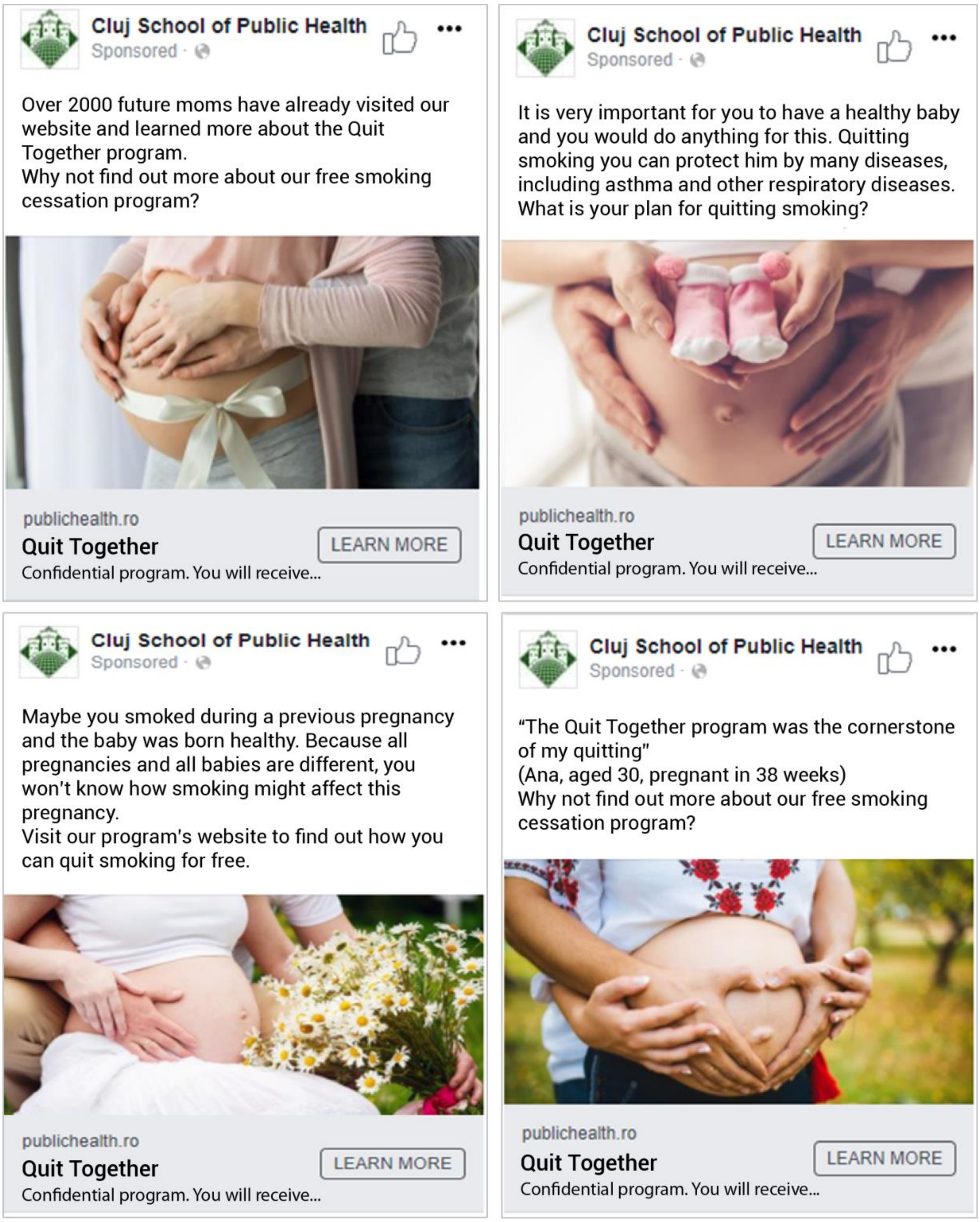

Figure S6. Quit Together Facebook ads developed based on behavioral economics insights.
